# Therapeutic potential of human minor salivary gland epithelial progenitor cells in liver regeneration

**DOI:** 10.1038/s41598-017-11880-z

**Published:** 2017-10-05

**Authors:** Chen Zhang, Yan Li, Xiang-yu Zhang, Lei Liu, Hai-zhou Tong, Ting-lu Han, Wan-di Li, Xiao-lei Jin, Ning-bei Yin, Tao Song, Hai-dong Li, Juan Zhi, Zhen-min Zhao, Lin Lu

**Affiliations:** 1 Department No.16, Plastic Surgery Hospital, Chinese Academy of Medical Sciences and Peking Union Medical College, 33 Ba Da Chu Road, Beijing, 100144 P.R. China; 2 International Medical Plastic and Cosmetic Centre, China Meitan General Hospital, 29 Xi Ba He Nan Li Road, Beijing, 100028 P.R. China; 3 Department of Breast Surgery, The First Affiliated Hospital of Zhengzhou University, 1 Jian She East Road, Zhengzhou, Henan Province 450003 P.R. China; 4 Department of Cleft Lip and Palate, Plastic Surgery Hospital, Chinese Academy of Medical Sciences and Peking Union Medical College, 33 Ba Da Chu Road, Beijing, 100144 P.R. China; 5 Department of Anesthesia, Plastic Surgery Hospital, Chinese Academy of Medical Sciences and Peking Union Medical College, 33 Ba Da Chu Road, Beijing, 100144 P.R. China; 6 Department of Stomatology, Beijing Children’s Hospital, Capital Medical University, 56 Nan-Li-Shi Road, Beijing, 100045 P.R. China; 7 Department of Plastic and Reconstructive Surgery, Shanghai Ninth People’s Hospital, No. 639 Zhizaoju Road, Shanghai, 200011 P.R. China

## Abstract

Liver disease is a serious problem affecting millions of people with continually increasing prevalence. Stem cell therapy has become a promising treatment for liver dysfunction. We previously reported on human minor salivary gland mesenchymal stem cells (hMSGMSCs), which are highly self-renewable with multi-potent differentiation capability. In this study, keratinocyte-like cells with self-regeneration and hepatic differentiation potential were isolated and characterized, and named human minor salivary gland epithelial progenitor cells (hMSG-EpiPCs). hMSG-EpiPCs were easily obtained *via* minor intraoral incision; they expressed epithelial progenitor/stem cell and other tissue stem cell markers such as CD29, CD49f, cytokeratins, ABCG2, PLET-1, salivary epithelial cell markers CD44 and CD166, and the Wnt target related gene LGR5 and LGR6. The cells were induced into functional hepatocytes *in vitro* which expressed liver-associated markers ALB, CYP3A4, AAT, and CK18. Upon transplantation *in vivo*, they ameliorated severe acute liver damage in SCID mice caused by carbon tetrachloride (CCl_4_) injection. In a two-thirds partial hepatectomy mouse model, the transplanted cells survived at least 4 weeks and exhibited hepatic potential. These findings demonstrate that hMSG-EpiPCs have potential as a cellular therapy basis for hepatic diseases, physiological and toxicology studies and regenerative medicine.

## Introduction

Liver dysfunction, which may progress to fulminant or chronic liver failure, is a serious healthcare problem worldwide. In some cases, it even deteriorates into end-stage liver disease. Thus far, orthotopic liver transplantation (OLT) has been considered to be the ultimate therapeutic approach. However, with obvious drawbacks such as organ scarcity, postoperative complications, and life-long requirement for immunosuppressive medication, traditional treatments should be advanced by novel strategies. In recent years, cell-based therapy to promote hepatic self-regeneration has become a promising alternative for treatment of liver failure^1–4^.

Currently, adult stem/progenitor cells are attracting significant interest in tissue engineering and regenerative medicine. These cells exist within various tissues^5–7^. Previous studies mainly focused on application of mesenchymal stem cells to the treatment of endodermal tissues^8,9^. Recent studies have attempted to isolate an appropriate source of endodermal adult stem/progenitor cells, such as salivary glands, pancreas, and liver^10–15^. Cells from such sources could facilitate the investigation of endodermal organ developmental processes and help to realize cell therapy and tissue engineering-based reconstruction.

Major salivary gland-derived epithelial cells are well-studied^11,16–21^ and have been successfully differentiated into both salivary gland and endodermal cell lineages, such as hepatocytes and pancreatic beta cells^11,22,23^. However, the major salivary glands do not seem to be an ideal and safe option for stem/progenitor cell harvest because of the invasive procedure to the donor site, whereas the minor salivary glands located in oral mucosa appear to be an optimal source with easy access and less invasion^24^. Therefore, we hypothesized that the minor salivary glands might contain epithelial stem/progenitor cells with ability to differentiate into other endodermal lineages. Previously, we reported the existence of both human minor salivary gland mesenchymal stem cells (hMSGMSCs) and epithelial progenitor cells (hMSG-EpiPCs) derived from human labial salivary glands^25,26^. This present study further details the phenotypes and characterization of the epithelial progenitor cells.

## Results

### Isolation and self-renewal capacity of hMSG-EpiPCs

**Figure 1:**
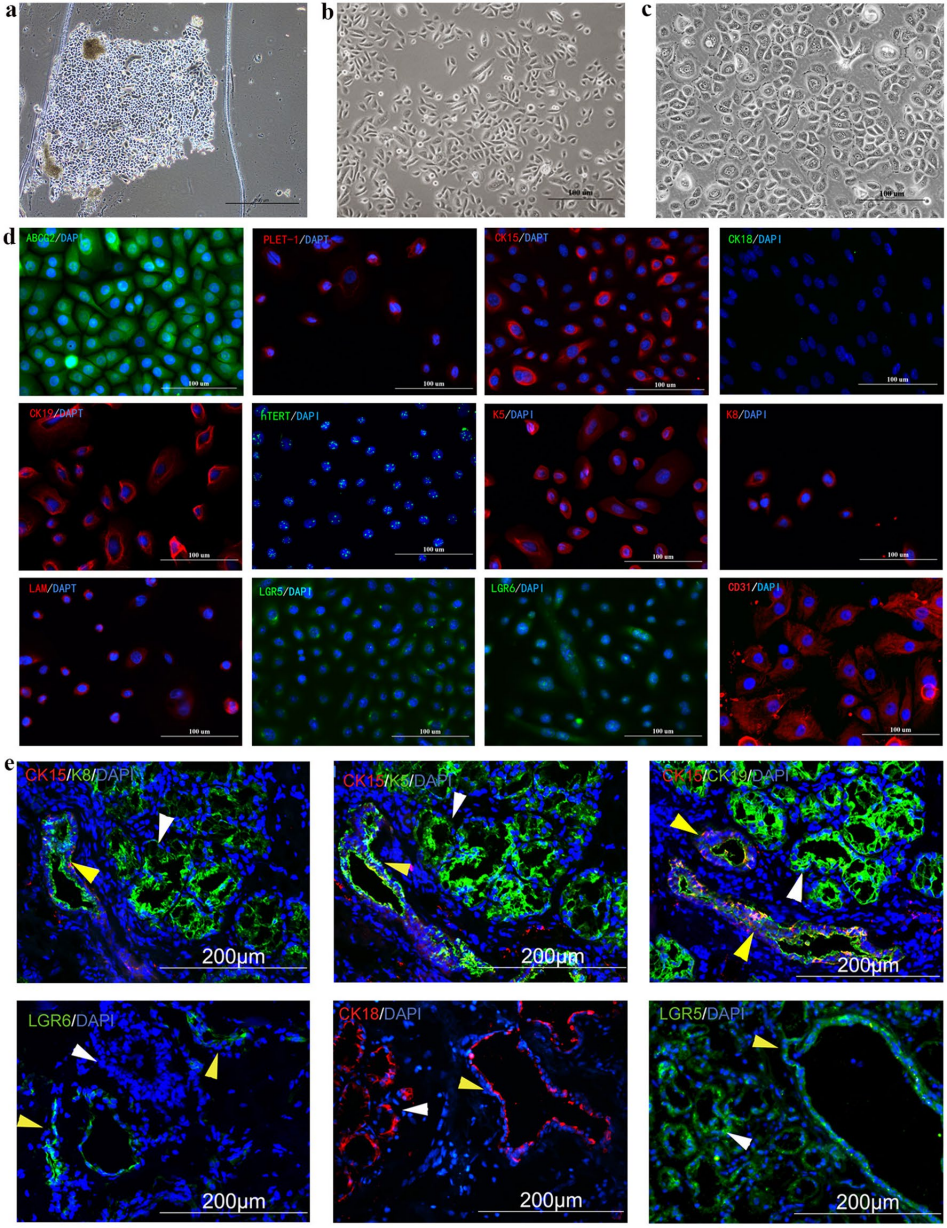
Culture and Characterization of hMSG-EpiPCs. (**a**) Primary culture for hMSG-EpiPCs at day 5. (**b**) Expanded hMSG-EpiPCs of passage 3. (**c**) Expanded hMSG-EpiPCs of passage 25. (**d**) Immunostaining of expanded hMSG-EpiPCs of passage 5. (**e**) Immunostaining of hMSG tissues (Yellow arrows: excretory duct, white arrows: acinus).

Tissue explant method was used for primary cell culture after human minor salivary glands (hMSG) were surgically harvested. After 5–10 days of culture, keratinocyte-like cells migrated from the tissue explants (Fig. 1a). The first passage usually occurred at day 7–10, when the cells around the tissue explant reached 80%-90% confluence. Normally, we obtained approximately 5 × 10^6^ homogeneously shaped cells at passage 3 (Fig. 1b). During the passaging, the cell growth and morphology did not show obvious changes until 25 passages (Fig. 1c). MTT and colony formation assays were performed for the cells at passage 5 to test their proliferation ability. The cell population doubling time was 70.85 hours on average, whereas the efficiency of colony formation was 12.3% ± 2.1%. Such results suggest the existence of a population of epithelial progenitor cells derived from hMSGs that could maintain high proliferation capacity during culture *in vitro*; we named the population as human minor salivary gland epithelial progenitor cells (hMSG-EpiPCs).

### Identification and characterization of hMSG-EpiPCs

Flow cytometry analysis of these keratinocyte-like cells at passage 5 demonstrated that the expanded cells highly expressed the epithelial lineage-related markers CD29, CD49f, and CK-KIT (CK14, CK15, CK16, and CK19) as well as the salivary epithelial cell markers CD44 and CD166 (Table 1, Supplementary Fig. S1a). In addition, the cells were negative for the mesenchymal stem cell markers CD90, CD105, CD106, and STRO-1, indicating non-mesenchymal origin. Interestingly, in contrast to other epithelial stem/progenitor cells, these cells included a population positive for the hESC marker SOX2, suggesting consistency with other stem cell types.

**Table 1:** Flow cytometry analysis of hMSG-EpiPCs.

	Average	s.d.
**Epithelial lineage-related markers**		
CD29	99.23%	0.76%
CD49f	93.61%	6.07%
CK-KIT	95.03%	4.90%
**Salivary epithelial cell markers**		
CD44	83.07%	6.44%
CD166	99.83%	0.12%
**Mesenchymal stem cell markers**		
CD90	2.08%	1.36%
CD105	2.34%	0.37%
CD106	1.87%	0.86%
STRO-1	1.64%	0.84%
**Hematopoietic lineage-related markers**		
CD117	0.44%	0.45%
**Embryonic stem cell markers**		
SOX2	67.03%	4.67%
SSEA-1	5.13%	1.73%
NANOG	1.11%	0.86%
OCT4	1.99%	1.28%
**Other markers**		
ALB	0.58%	0.22%

Averaged percentage of flow cytometry analysis of hMSG-EpiPCs at passage 5 for the cell markers are shown in the table. Standard deviation is shown as s.d.

The phenotypes of both hMSG-EpiPCs and hMSG tissues were determined via immunofluorescence staining (Fig. 1d and e). Positive expression of the epithelial lineage-related markers CK15, CK19, ABCG2, and PLET-1 was observed. We also detected human telomerase reverse transcriptase (hTERT), the thymic epithelial progenitor cell markers K5 and K8, the endothelial cell marker CD31, and the basal lamina protein laminin in hMSG-EpiPCs and minor salivary gland tissues. Furthermore, the Wnt pathway target genes LGR5 and LGR6, markers of several lineages of stem/progenitor cells, including liver-derived stem cells, were also expressed. Tissue immunofluorescent staining showed expression of the epithelial lineage-related markers CK18, CK19, K5, and K8 and the Wnt target gene related LGR5 in the excretory duct and acinus; CK15-positive and LGR6-positive cells were located only in the excretory duct, indicating that these cells originated from the excretory duct of the hMSG.

These results demonstrate that hMSG-EpiPCs expressed phenotypes of epithelial stem/progenitor cells and other adult stem cell types, and suggest that these cells originated from the excretory duct part of the minor salivary glands.

### Hepatic induction of hMSG-EpiPCs ***in vitro***

**Figure 2:**
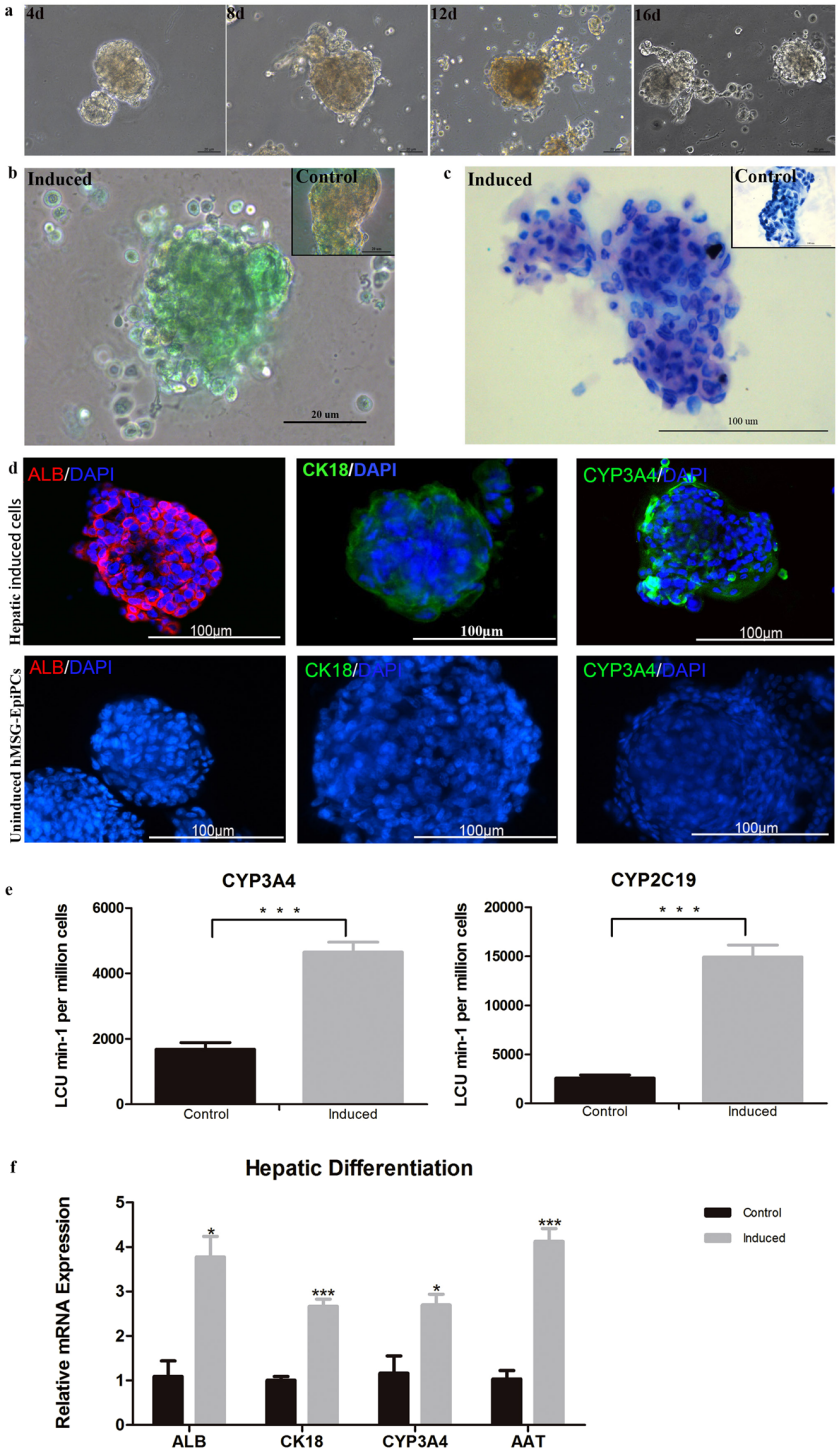
Hepatic differentiation of hMSG-EpiPCs *in vitro*. (**a**) Picture of induced hMSG-EpiPCs taken on day 4, 8, 12 and 16 from left to right. (**b**) Indocyanine green (ICG) uptake assay on day 16 of hepatic induction and the control group. (**c**) Periodic acid-Schiff (PAS) staining on day 16 of hepatic induction and the control group. (**d**) Immunostaining on day 16 of hepatic induced and uninduced cells for ALB, CK18 and CYP3A4 from left to right. (**e**) CYP450 activity analysis on day 16 of hepatic induction for CYP3A4 and CYP2C19. (**f**) Quantitative real-time PCR analysis of relative mRNA expression of hMSG-EpiPCs and induced hepatic cells on day 16. (***P < 0.01; n ≧ 3).

To induce hepatic differentiation of hMSG-EpiPCs *in vitro*, cells were cultured in 24-well plates containing thick Matrigel with conditioned medium for 16 days. hMSG-EpiPCs formed spheres in Matrigel during the induction procedure (Fig. 2a). After induction, the cells expressed a diverse panel of markers for hepatocytes including ALB, CYP3A4, and CK18 while the uninduced cells were negative (Fig. 2d). Gene expression analysis showed that expression of the hepatocellular markers ALB, CYP3A4, CK18, and AAT increased during differentiation (Fig. 2f). CYP450 activity, indocyanine green (ICG) uptake, and periodic acid-Schiff staining tests were performed to further validate function of the induced hepatocytes(Fig. 2b,c and e). The results suggested that successful hepatic differentiation was achieved *in vitro*, indicating the hepatic differentiation potential of hMSG-EpiPCs.

### Therapeutic potential of hMSG-EpiPCs in CCl_4_-induced acute fulminant liver failure

**Figure 3:**
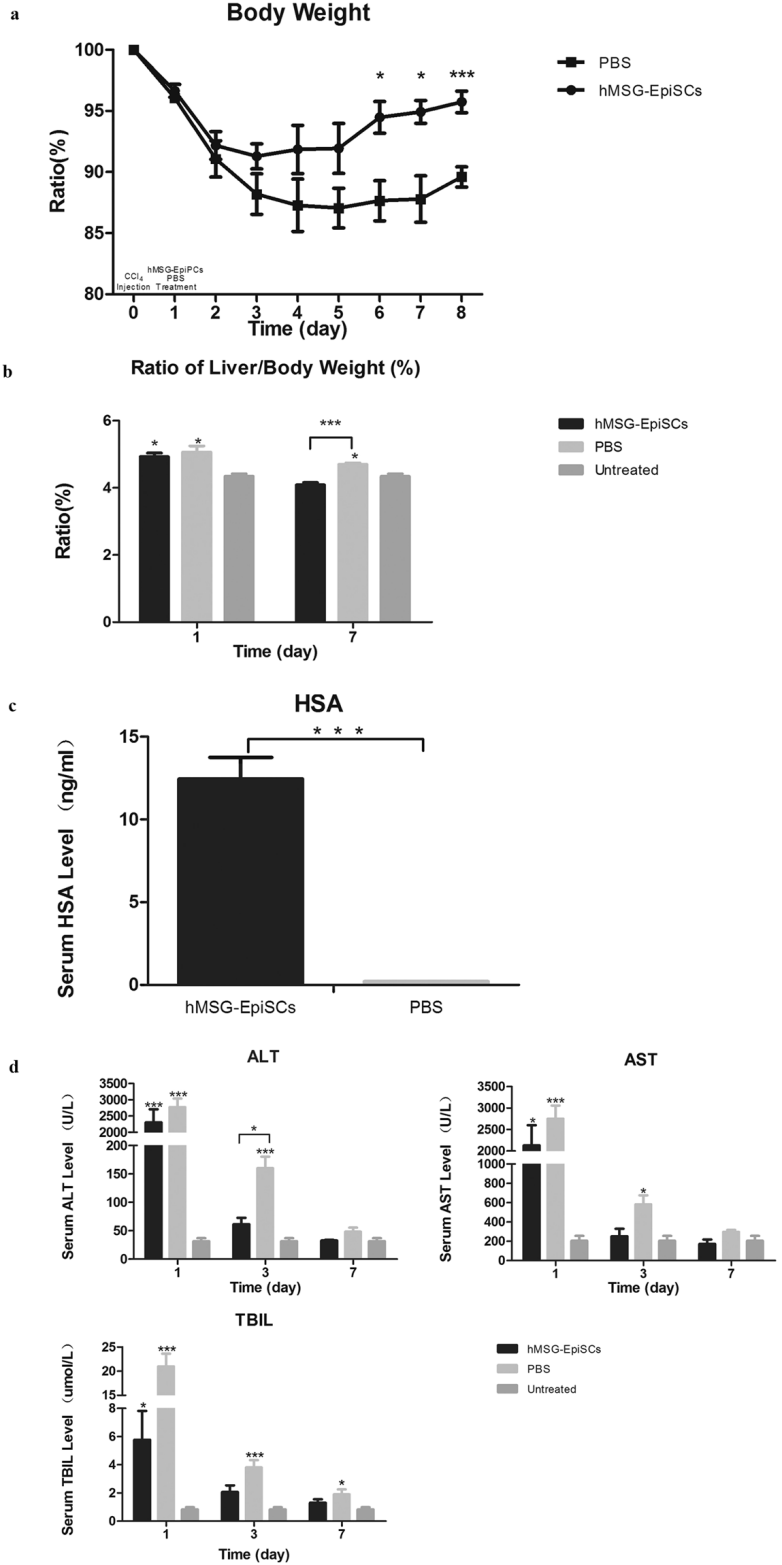
hMSG-EpiPCs treatment promotes liver regeneration after acute injury. (**a**) Changes in body weight of CCl_4_-injected mice show that hMSG-EpiPCs treatment group restored body weight faster than control group. (**b**) The ratio of liver weight to body weight of the hMSG-EpiPCs treatment mice was lower than that of the PBS-treated mice. (**c**) HSA could be detected in hMSG-EpiPCs treatment group. (**d**) Serum ALT,AST and TBIL level increased in all mice 2 day after CCl_4_ injection, and decreased faster in hMSG-EpiPCs treatment group than that of control mice. (*P < 0.05; ***P < 0.01, n ≧ 3).

To test whether hMSG-EpiPCs could promote liver regeneration *in vivo*, we performed cell transplantation in the animal model of fulminant hepatic injury. By injection of CCl_4_ acute liver injury was triggered in severe combined immunodeficient (SCID) mice. The cell-treated group was transplanted with hMSG-EpiPCs, whereas the sham group was treated with PBS, and no disease model or treatment was applied in the control group. Starting at day 1 after CCl_4_ injection, the body weight (BW) of the mice in the sham group kept decreasing until day 5 as a result of inflammatory hepatomegaly. Three mice (30% mortality) died 2 days after CCl_4_ injection. While all mice treated with hMSG-EpiPCs survived and recovered from liver damage in a shorter time period, along with a lower ratio of liver weight to BW (Fig. 3a and b).

Human serum albumin (HSA) was detected in the serum of treated mice 2 weeks after injection of the cells (Fig. 3c), whereas it was scarcely detected in the sham group. In addition, in both disease model groups, a dramatic increase in serum alanine transaminase (ALT), aspartate transaminase (AST), and total bilirubin (TBIL) was detected 2 days after CCl_4_ injection (1day after hMSG-EpiPC/PBS treatment), indicating severe acute liver damage. ALT, AST, and TBIL levels of animals in the experimental-group decreased to baseline significantly faster than the levels in the sham group (Fig. 3d).

**Figure 4:**
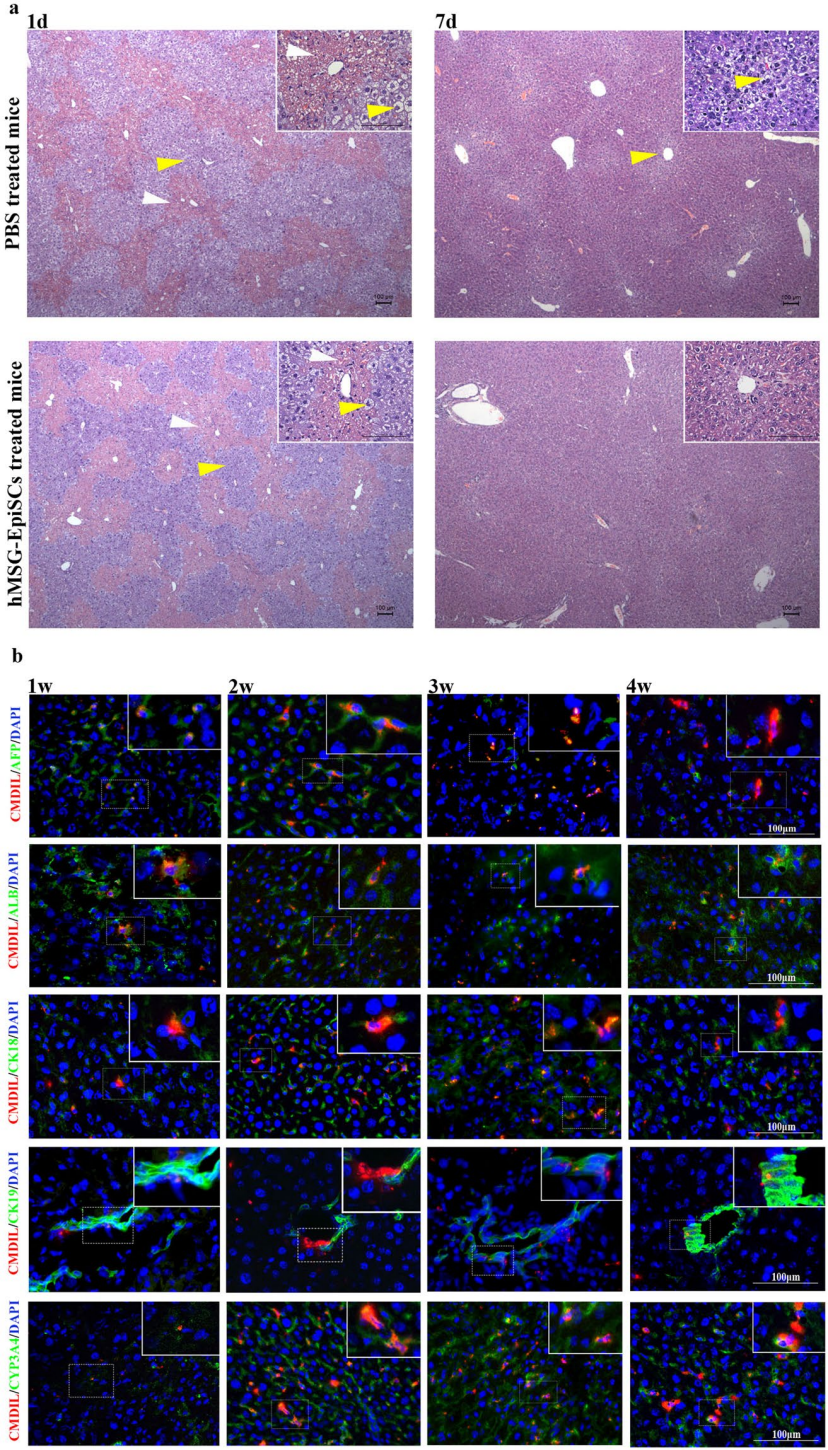
Hepatic differentiation of hMSG-EpiPCs *in vivo*. (**a**) H&E staining of mice liver section on day 1 and 7 post PBS and hMSG-EpiPCs injection (White arrows: erythrocyte diapedesis, yellow arrows: diseased hepatocytes). (**b**) Immunostaining 1, 2, 3 and 4 weeks after hMSG-EpiPCs transplantation from left to right, green fluorescence signal for AFP, ALB, CK18, CK19 and CYP3A4 from top to bottom, and red for CM-Dil labeling.

Histological analysis revealed that hepatocyte necrosis occurred 2 days after injection of CCl_4_ in both hMSG-EpiPC- and PBS-treated groups (24 h after hMSG-EpiPC or PBS injection) (Fig. 4a). Hepatocytes presented as vacuolar, hydropic, karyorrhexic, and karyolytic, and erythrocyte diapedesis was also observed. At 8 days post CCl_4_ injection (7 days post hMSG-EpiPC or PBS injection), both groups had recovered from acute liver damage, but diseased hepatocytes still could be observed in the PBS-treated group while no such cells existed in the hMSG-EpiPC-treated group, suggesting enhanced liver regeneration in the hMSG-EpiPC-treated mice.

To test the survival of the cells, we transplanted hMSG-EpiSCs labelled by cell-tracker CM-Dil (Supplementary Fig. S1b) to this mouse model. Liver tissues were harvested two weeks after the transplantation. Immunofluorescence assays showed that several CM-Dil-positive cells survived *in vivo* and expressed for AFP, ALB and CK18 (Supplementary Fig. S1c).

### Therapeutic potential of hMSG-EpiPCs in a mouse model of partial hepatectomy

To in detail validate the survival of hMSG-EpiPCs *in vivo*, we further transplanted hMSG-EpiSCs labelled with cell-tracker CM-Dil in a mouse model of two-thirds partial hepatectomy. We euthanized cell-treated mice every week post transplantation and harvested the liver tissues for immunofluorescence assays (Fig. 4b) and the liver tissues of normal SCID mice were stained as control (Fig. S1d). One week post injection, few CM-Dil-positive cells were positive for ALB, AFP, CYP3A4, CK18, or CK19. More CM-Dil-positive cells expressed ALB, AFP, CYP3A4, or CK18 two weeks after injection. Cells double-positive for CK19 and CM-Dil appeared surrounding the portal tract. At 3 weeks after transplantation, ALB-positive cells formed cluster involving several CM-Dil-labelled cells. Count of AFP-positive cells decreased. More CM-Dil-labelled cells occurred around the portal tract, and some of them were also positive for CK19, indicating participation of the transplanted hMSG-EpiPCs in the reconstruction of the intrahepatic bile duct. CYP3A4 and CK18 were strongly expressed at this time, with many of the positive cells double-positive with CM-Dil. At 4 weeks after transplantation, few AFP-positive cells showed double positivity. We found abundant CYP3A4/CM-Dil, ALB/CM-Dil and CK18/CM-Dil co-positive cells, as well as CK19/CM-Dil co-positive cells surrounding the portal tract (Table 2). No evidence of hepatic tumour formation in the hMSG-EpiPC-treated livers was found at any time point.

**Table 2:** Immunofluorescence Analysis of hMSG-EpiPCs Transplantation *in vivo*.

Antibody	1 Week	2 Weeks	3 Weeks	4 Weeks
AFP	16.4 ± 3.4	25.4 ± 4.5	13.6 ± 3.5	4.8 ± 3.1
ALB	6.5 ± 2.9	12.8 ± 3.9	12.5 ± 4.7	13.5 ± 3.8
CK18	5.0 ± 2.2	14.0 ± 4.2	20.5 ± 4.5	20 ± 3.8
CK19	1.8 ± 0.8	2.8 ± 1.1	3.0 ± 1.3	2.5 ± 0.5
CYP3A4	6.5 ± 2.9	20.8 ± 2.4	28.5 ± 4.1	27.3 ± 3.8

Averaged number of injected CM-Dil-labeled double positive cells in different time after the transplantation are shown in the table.

## Discussion

The lack of an easily accessed population of human adult endodermal stem/progenitor cells with multi-differentiation potential is a challenge for cell therapy and regenerative medicine. The hMSGs locating in the oral mucosa may be promising candidates as cell source for their easy accessibility and adequate cell quantity^24^. In a previous study, we reported the existence of hMSGMSCs with phenotypes of embryonic and mesenchymal stem cells^26^. These hMSGMSCs are highly self-renewable and have multi-potent differentiation potential. The hMSG-EpiPCs we reported here were also isolated from primary cultured hMSG. However, these keratinocyte-like hMSG-EpiPCs showed a different shape and phenotype that suggested an epithelial lineage. These hMSG-EpiSCs showed high efficiency in endodermal differentiation *in vitro*, which inspired us to further research their therapeutic potential in liver regeneration.

In the current study, cells expressing CK18, CK19, K5, K8, and/or LGR5 were found in both the excretory duct and acinus, whereas expression of CK15 and LGR6 was observed only in the excretory duct. Thus, we propose the existence of an epithelial progenitor cell population mainly derived from the hMSG excretory duct; these oval-shaped keratinocyte-like cells were named hMSG-EpiPCs.

In contrast to mesenchymal stem cells, there are no universal standard phenotypic markers for stem/progenitor cells of epithelial-lineage. Various markers are used to mark epithelial stem/progenitor cells with various origins. Flow cytometry analysis detected high expression of the epithelial lineage-related markers CD29, CD49f, and CK-KIT in hMSG-EpiPCs, together with markers previously identified in salivary-related cells such as CD44 and CD166^27^. ABCG2, which has been reported to be positively expressed in progenitor cells isolated from mouse submandibular glands^28^, was also found to be expressed in epithelial progenitor cells isolated from hMSGs. Immunofluorescence staining revealed that hMSG-EpiPCs expressed hTERT, which exerts control over cell proliferation^29^. Additionally, the thymic epithelial progenitor cell markers K5, K8, and PLET-1 were expressed in hMSG-EpiPCs and in minor salivary gland tissue^30–32^. Cytokeratins are proteins found in the intracytoplasmic cytoskeleton of epithelial tissue that were previously reported to identify mouse postnatal mammary gland epithelial cells^33^, human hair follicle stem cells^34^, and human liver progenitor cells^35^. In addition, Lombaert IM previously reported that salivary gland cells isolated from murine submandibular glands were positive for CD117, while hMSG-EpiPCs were negtive^36^. These observations help to confirm the existence of hMSG-EpiPCs.

In addition to these epithelial-lineage markers, hMSG-EpiPCs were also positive for the endothelial cell marker CD31, which suggest their applicability to vascular tissue engineering. Expression of the basal lamina protein laminin was also observed. In rat salivary glands, ligation of main ducts caused the disappearance of acinar cells and proliferation of ductal cells around laminin^+^ cells, which suggests activation of tissue progenitor cells in a damaged state^24^. Cultured hMSG-EpiPCs included a population of cells positive for the hESC marker Sox2,which maintains pluripotency of hESCs and regulates formation of various epithelial tissues during foetal development^37,38^, which may explain the high proliferation ability of hMSG-EpiPCs.

Additionally, the Wnt target genes LGR5 and LGR6, which are markers of several stem/progenitor cells^14,39–46^, were also positively expressed, especially LGR5, which is a liver-derived stem cell marker^13,15^. This LGR5 expression supports the potential use of hMSG-EpiPCs as a cellular basis for regenerative liver medicine.

In the present study, the hepatic cells differentiated from hMSG-EpiSCs expressed ALB, CK18, and CYP3A4. The expression of ALB, CK18, and CYP3A4 demonstrated fully mature hepatocyte function. In addition, Q-PCR showed that expression of AAT increased during differentiation. AAT is mainly produced in the liver and protects the lungs against proteolytic damage^47^. However, expression of AAT was not observed on immunofluorescent staining, and this result thus needs further study. Cell functionality was validated through analyses of CYP450 activity, ICG uptake, and periodic acid-Schiff staining, indicating that fully functional hepatocytes were acquired from hMSG-EpiPCs.

To further test the clinical potential of hMSG-EpiPCs, we used different animal models to validate the therapy effect. CCl_4_ is a model substance for elucidating hepatotoxic mechanisms such as hepatocellular death, fibrosis, fatty degeneration, and carcinogenicity^48^. After injection of 20% CCl_4_, the levels of ALT, AST, and TBIL dramatically increased as a result of enzyme release caused by massive hepatocyte damage, indicating acute and massive death of hepatocytes, which also caused the body weight of the mice to decrease. Hepatomegaly occurred, according to the pathological sections. In the PBS-treated group, 3 mice (30% mortality) died 2 d after CCl_4_ injection. The ALT, AST, and TBIL levels decreased faster in the hMSG-EpiPC-treated mice than in their PBS-treated counterparts, along with faster body weight increases, faster hepatocyte regeneration, a higher survival rate, and lower ratio of liver weight to BW. Moreover, the detection of HSA in the serum of hMSG-EpiPC-treated mice and the immunofluorescence assays result two weeks after cell injection implied proliferation and hepatic differentiation of transplanted hMSG-EpiPCs. The mouse model of two-thirds partial hepatectomy is a relatively “clean” model of hepatic damage, with no massive hepatocyte death and mild inflammation; this model does not cause inflammatorycellinfiltration or negatively impact the engraftment of transplanted stem/progenitor cells^49^. Weber *et al*. reported that after 70% hepatectomy, liver regeneration was activated immediately, and the weight of the remaining liver had increased to a normal level 20 d after the surgery^50^. Our result shows that after hMSG-EpiPC injection, the number of ALB/CM-Dil, CK18/CM-Dil and CYP3A4/CM-Dil double-positive cells increased daily, whereas AFP/CM-Dil double-positive cells occurred 1 week after transplantation, reached their highest level 2 weeks after transplantation, and then gradually decreased in number, which suggests both proliferation and hepatic differentiation of the transplanted hMSG-EpiPCs. Both differentiated and non-differentiated hMSG-EpiPCs expressed CK19 *in vitro*, which is a marker of both the minor salivary gland excretory duct and the bile duct. After injection of hMSG-EpiPCs, most of the CM-Dil-positive cells were negative for CK19; a few positive cells occurred only in the portal area. Therefore, we speculate that hMSG-EpiPCs promote liver regeneration through two major mechanisms. One is by differentiating into hepatocytes expressing ALB, AFP, CK18, and CYP3A4. The other is by migrating into the portal area and participating in the reconstruction of the intrahepaticbileduct. Therefore, hMSG-EpiPCs showed a positive effect in both disease models, indicating that they are a promising candidate for treatment of liver diseases.

## Conclusion

In the current study, we isolated and characterized a population of epithelial progenitor cells from hMSGs. These cells have self-renewal and hepatic differentiation capacity. We also demonstrated the ability of these cells to ameliorate liver damage through transplantation in acute liver injury models. These cells have potential for hepatic differentiation. Our study highlights the importance of hMSG-EpiPCs in hepatic disease modelling, physiological and toxicology studies, regenerative medicine, and clinical therapy.

## Method

### Ethical Statement

All experimental procedures were approved by Institutional Review Board of the Plastic Surgery Hospital, Peking Union Medical College and Animal Care and Use Committee of the Plastic Surgery Hospital, Peking Union Medical College, and were in accordance with the Declaration of Helsinki (2013). Tissues containing salivary glands were harvested from consenting donor patients with cleft lip and palate deformity during surgery, and none of these patients was diagnosed with salivary gland diseases. All these patients have signed the Specimen Collection Informed Consent.

### Mice

Female severe combined immune deficiency (SCID/Beige) mice aged 6–8 weeks were provided by the Academy of Military Medical Sciences (Beijing, China).

### Human Salivary Gland Tissue and Cell Culture

Tissues specimens containing salivary glands were transported in 15-mm centrifugetubes filled with regular medium (Dulbecco’s modified Eagles medium (DMEM, HyClone), supplemented with 1% penicillin and streptomycin (5,000 UmL^−1^) (Sigma), 1% GlutaMAX supplement (Gibco), and 10% foetal bovine serum (Gibco)). When isolating hMSG-EpiPCs for primary culture, excess connective tissues were removed by scissors. The glands were cut into small pieces no larger than 1 mm^3^ and placed at the base of the culture flask and then incubated for 3 to 4 h, before 5 mL of keratinocyte medium (ScienceCell) was added.

The medium was first changed at day 5, and then changed every 3 days. Cells at 80% confluence were harvested with 0.25% trypsin and re-seeded in a cell culture dish. The media were changed every 3 days. Cells were passaged using 0.25% trypsin (HyClone) at 80% confluence in a 1:3 split ratio.

### MTT Assay

The MTT assay (Sigma) was performed to determine the proliferation ability of the cells. At 80% confluence, cells at passage 5 were trypsinized and transferred to a 96-well plate at a density of 2 × 10^3^ per well. MTT solution was added to each well of the 96-well culture plate, which was then incubated at 37 °C for 4 h. After the MTT solution was removed, samples were dissolved in DMSO (Sigma) and transferred to new 96-well plates. The absorbance of each well was measured at a wavelength of 490 nm for 7 continuous days. The cell population doubling time was calculated as previously described^26^.

### Cell Colony Formation Assay

Cells at passage 5 were trypsinized and seeded in a 60-mm dish atdensity of 200 cells and then cultured for 2 weeks. The media were changed every 3 days. The surviving colonies (>50 cells/colony) were counted using crystal violet staining.

### Flow Cytometry

Samples at passage 5 were collected from 60-mm dishes at 80% confluence (1 × 10^6^ cells). The cells were washed, fixed and incubated with antibodies as previously described^26^. We used a FACSAria II system (BD) for analyses. For each marker, 3 independent biological experiments were performed. FlowJo 7.6.1 software (Tree Star Inc. OR) was used to analyse the acquired data.

### Hepatic Differentiation

We used a thick Matrigel culture method to induce hepatocytes according to the manufacturer’s instructions (BD). Cells at passage 4 and 80% confluence were trypsinized and counted. Then, 1 × 10^4^ cells were resuspended in liver induction medium, added to 24-well plates/chambers, and further cultured under 5% CO_2_ at 37 °C in a cell incubator. The liver induction medium was changed every 3 days for a period of 16 days. It consisted of KM basal medium (ScienceCell) supplemented with 1% ITS Liquid Media Supplement (Sigma), 10 ngmL^−1^ IGF (Peprotech), 20 ngmL^−1^ EGF (Peprotech), 50 μgmL^−1^ BPE (ScienceCell), 20 ngmL^−1^ HGF (Peprotech), 10 mM nicotinamide (Sigma), 100 nM dexamethasone (Sigma), and 100 UmL^−1^ penicillin-streptomycin (Sigma).

### Transplantation Assay

To determine whether transplanted MSG-hEpiPCs could promote liver regeneration *in vivo*, liver damage was produced in the mice by intraperitoneal injection of 10 mlkg^−1^ CCl_4_ dissolved in olive oil (1:4). At 24 h after injection, 150 μL suspensions of hMSG-EpiPCs at passage 5 labeled with CM-Dil were injected via the caudal vein(approximately including 2 × 10^6^ cells). The body weight of each mouse was measured, and liver tissues and serum were collected 1 day, 3 days, 1 week, and 2 weeks after hMSG-EpiPC injection for assays.

To test the proliferation of transplanted hMSG-EpiPCs *in vivo*, a mouse model of two-thirds partial hepatectomy was established as previously described, after which hMSG-EpiPCs at passage 5 were labelled with CM-Dil and injected immediately, as described above. The liver tissues were collected at 1 week, 2 weeks, 3 weeks, and 1 month after hMSG-EpiPC injection for immunofluorescence staining. The quantity of CM-Dil-labeled double positive cells in the immunofluorescence staining sections was measured under a Nikon TE2000-S microscope. Three researchers blinded to the groups counted the number in 15 randomized filelds on each section in 40 × magnification.

### Immunohistochemistry and Immunofluorescence

Slides of hMSGs, cells, and liver tissues were fixed for 15 min in precooled 4% paraformaldehyde. The hMSG sections were stained with hematoxylin and eosin. For immunofluorescence, slides of tissues and cells were stained using standard techniques as described previously^26^. Images were acquired using a Nikon TE2000-S microscope, and an Olympus laser scanning confocal microscope.

### Quantitative Reverse Transcriptase–polymerase Chain Reaction

Total RNA was obtained from cultured cells using the RNeasy Mini kit (Qiagen) and reverse-transcribed using M-MLV (Invitrogen) according to the manufacturer’s protocol. Real-time PCR was performed using SYBR Green I Master (Roche) on a LightCycler 480 system (Roche Molecular Systems, CA). Expression levels of desired genes were normalized to those of GAPDH as a reference gene. The samples were collected from three independent biological replicates.

### CYP450 Activity Analysis

The CYP3A4 and CYP2C19 ability of the induced cells was measured by using P450-Glo assays (Promega catalogue nos. V9001 and V8881) according to the manufacturer’s protocol. The luminescence was read by an ELISA detector. Results are shown as LCU min^−1^ normalized to 10^6^ cells.

### Periodic Acid-Schiff Staining

Periodic acid-Schiff staining (Sigma-Aldrich) was performed according to the manufacturer’s protocol.

### Indocyanine Green Uptake

Cells were incubated with indocyanine green (ICG, CardioGreen; Sigma-Aldrich, 12633) in DMEM (1 mgmL^−1^) for 30 min in and 37 °C under 5% CO_2_ in a cell incubator. Subsequently, the cells were washed three times with PBS. ICG uptake was visualized using a Nikon TE2000-S microscope.

### Assessment of Liver Function

Blood serum samples were taken for assessment, and the amount of AST (aspartate transaminase; Roche), ALT (alanine transaminase; Roche), and totalbilirubin (TBIL; Roche) was estimated using commercial kits in accordance with the manufacturer’s protocol.

### HSA ELISA

The amount of HSA (human serum albumin) in serum was detected using a human-specific ALB ELISA kit (catalogue no. EA3201-1; Assaypro), according to the manufacturer’s instructions.

### Statistical Analysis

All statistical analyses were performed using the SPSS software package. Statistical significance was defined as P < 0.05. Two-tailed student’s *t*-test was performed to compare two groups.

## Electronic supplementary material

Supplementary Information
