# Supplementary material for: Therapeutic potential of human minor salivary gland epithelial progenitor cells in liver regeneration
Source: Sci Rep. 2017 Oct 5;7:12707. doi: 10.1038/s41598-017-11880-z (PMC5629247; doi:10.1038/s41598-017-11880-z)
Supplement: Supplementary file 1 — Supplementary Information [file 41598_2017_11880_MOESM1_ESM.pdf]

Article Title: Therapeutic potential of human minor salivary gland epithelial progenitor cells in liver regeneration

Authors:

Chen Zhang[1, #] E-mail address: nightglow123@gmail.com

Yan Li[2] E-mail address: s2007148@126.com

Xiang-yu Zhang[3] E-mail address: nchot@163.com

Lei Liu[4] E-mail address: ssydll@126.com

Hai-zhou Tong[4] E-mail address: 838472562@qq.com

Ting-lu Han[4] E-mail address: 1634877010@qq.com

Wan-di Li[4] E-mail address: wandili@hotmail.com

Xiao-lei Jin[1] E-mail address: jinyuyizxyy@126.com

Ning-bei Yin[4] E-mail address: ningbeiyin@gmail.com

Tao Song[4] E-mail address: gxstst@126.com

Hai-dong Li[4] E-mail address: 93041992@qq.com

Juan Zhi[5] E-mail address: hnzhijuan@163.com

Zhen-min Zhao[4, 6, *] E-mail address: zhaozhenmin0098@163.com

Lin Lu[7, *] E-mail address: lulin_12345@163.com

[#]First Author, [*]Co-corresponding Author.

Correspondence and requests for materials should be addressed to Zhenmin Z (zhaozhenmin0098@163.com) and Lin L (lulin_12345@163.com).

Supplementary Table.1 Product ID of Antibodies

Immunofluorescence Staining Antibody	Product ID
ABCG2	Abcam, ab24115
PLET1	Millipore, MAB4416
CK15	Abcam, ab93944
CK18	Abcam, ab7797
CK19	Abcam, ab52907
hTERT	Abcam, ab111584
K5	Sigma, SAB4501651
K8	Sigma, SAB4501653
Laminin	Abcam, ab49726
LGR5	Abcam, ab75732
LGR6	Abcam, ab126747
CD31	Abcam, ab76533
ALB	Abcam, ab137885
CYP3A4	Sigma, SAB5300118

Flow Cytometry Antibody	Product ID
CD29	Biolegend, 303015
CD49f	Biolegend, 313607
CK-KIT	BD, 550953
CD44	Biolegend, 103017
CD166	Biolegend, 343903
STRO1	Biolegend, 340103
SOX2	Biolegend, 656108
ALB	Abcam, ab137885
CD117	Biolegend, 313213

CD90	Biolegend, 328115
CD105	Biolegend, 323209
CD106	Biolegend, 305805
SSEA1	Biolegend, 125609
OCT4	Biolegend, 653703
NANOG	Biolegend, 674201

Supplementary Table.1 Product ID of Antibodies. The manufacture and product ID of antibodies we used in the immunofluorescence staining and flow cytometry analysis are shown in the table.

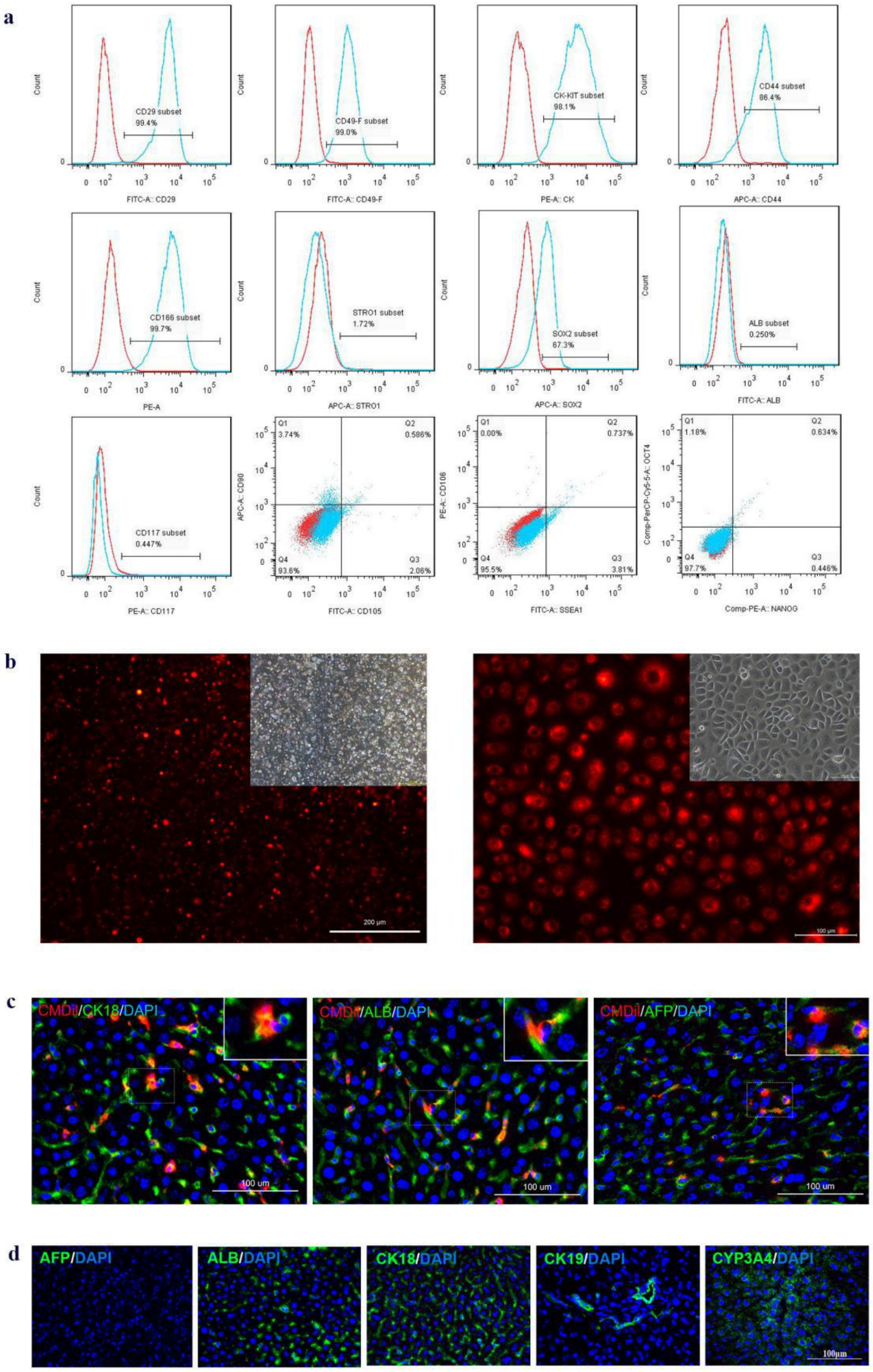

Supplementary Figure.1 (a) Representative flow cytometry analysis plots of hMSG-EpiPCs at passage 5. (b) Fluorescence photos of hMSG-EpiPCs immediately after labeled by CM-Dil (left) and cultured 2 days after the labelling (right). (c) Immunostaining 2 weeks after hMSG-EpiPCs transplantation, green fluorescence signal for AFP, ALB and CK18 from left to right, and red for CM-Dil labeling. (d)

Immunostaining of normal SCID mice the liver tissues, green fluorescence signal for AFP, ALB, CK18, CK19 and CYP3A4 from left to right.
